# Perceiving and expressing feelings through actions in relation to individual differences in empathic traits: the Action and Feelings Questionnaire (AFQ)

**DOI:** 10.3758/s13415-015-0386-z

**Published:** 2015-10-20

**Authors:** Justin H. G. Williams, Isobel M. Cameron, Emma Ross, Lieke Braadbaart, Gordon D Waiter

**Affiliations:** Royal Cornhill Hospital, Aberdeen, AB25 2ZH UK; University of Edinburgh Medical School, Edinburgh, UK; Aberdeen Biomedical Imaging Centre, University of Aberdeen, Aberdeen, UK; Clinical Research Centre, Division of Applied Health Sciences, University of Aberdeen, Royal Cornhill Hospital, Aberdeen, AB25 2ZH UK

**Keywords:** Embodied cognition, Motor, Emotion

## Abstract

Empathy is usually conceived of as independent of the non-verbal behaviors which mediate its experience, though embodied cognition theory predicts that individual differences in action representation will affect empathic traits. The “Actions and Feelings Questionnaire” (AFQ) was designed to capture individual differences in self-awareness of own and others’ actions, particularly those associated with feelings, which we predicted would correlate with levels of empathic traits. A pilot 30-item questionnaire included items on perceptual sensitivity to action, imitation, action imagery, and gestural and facial expression. It was completed by a sample of 278 adults (mean age 21.2 years; 189 females, 89 males) along with the 15-item Empathic Quotient (EQ) Questionnaire. Total scores on the final 18-item questionnaire showed strong internal coherence (Cronbach’s alpha of 0.81) and test-retest reliability (ICC=0.88), marked effect of sex and highly significant correlation with EQ. The questionnaire was administered to participants in an fMRI study investigating the neural correlates of facial imitation. Total AFQ score correlated with activity in somatosensory cortex, insula, anterior cingulate, and visual cortex. The AFQ shows promise as a brief and simple self-report measure sensitive to variability in the self-awareness of actions associated with feelings. It suggests that much of the variability of empathic traits in typical populations is accounted for by variance in this capacity. We suggest that being more empathic really is about being “touchy-feely,” and this questionnaire provides a novel measure of action-based empathy.

## Introduction

Nonverbal communication is critical to social functioning. It includes the regulation of eye contact, facial expression of emotion, and use of gesture. Intact social communicative abilities and in particular, the communication of emotion, require skills in expression and also sensitivity to their enactment by others. Impairments of nonverbal communication are a core feature of the diagnostic criteria for autism spectrum disorder and assessments of these abilities are key features of diagnostic instruments such as the Autism Diagnostic Observation Schedule and the Autism Diagnostic Interview (Lord et al., 2000; Rutter, Le Couteur, & Lord, 2000). The importance of nonverbal communication has been recognized in its making up of two constructs within the RDoC strategy (Morris & Cuthbert, 2012); http://www.nimh.nih.gov/research-priorities/rdoc/rdoc-constructs.shtml#production_nonfacial_communication). Nevertheless, paradigms for its research remain at early stages of development, perhaps because of the complex challenges concerned with the measurement of behaviors which have multiple degrees of freedom.

Because nonverbal behavior is so important for the communication of emotion between individuals, a question of particular interest is how nonverbal behavior relates to empathy. According to Decety (2011), the “term empathy is applied to various phenomena which cover a broad spectrum, ranging from feelings of concern for other people that create a motivation to help them, experiencing emotions that match another individual’s emotions, knowing what the other is thinking or feeling, to blurring the line between self and other.” De Vignemont and Singer (2006) describe empathy as a process whereby another person’s affective state evokes an isomorphic emotional state in the observer of which he or she is aware. Baron-Cohen and Wheelwright (2004) define it as a capacity to understand other people’s feelings and to respond to them appropriately. A distinction between the first two and the last definition is that empathy is defined either as being primarily dependent upon one individual evoking a feeling state in the other, or alternatively it relies on an individual “understanding” the other and responding appropriately. Notably, all these definitions define empathy as a phenomenon at an experiential level, and only Baron-Cohen and Wheelwright include an element whereby empathy is evidenced by enactment rather than what they report thinking or feeling, though even then it suggests a reliance on judgment rather than through their expression of empathy in the form on non-verbal behavior.

Embodied (or Grounded, Barsalou, 2008) theories of cognition (Niedenthal, Barsalou, Winkielman, Krauth-Gruber, & Ric, 2005) view the processes of knowledge use and acquisition as fundamentally grounded in their physical context and the brain’s modality-specific systems. This means that those cognitive operations that are heavily influenced by both action plans and emotional states are highly dependent upon those aspects of the brain that serve action plans and emotional states. With respect to empathy, grounded cognition theory argues that its cognitive computational mechanisms are grounded in the sensorimotor mechanisms that mediate its perception and expression. Hence, empathic function depends upon the capacity to “feel” the emotion, so implicating a critical and additional role for the somatosensory cortex (Bastiaansen, Thioux, & Keysers, 2009; Damasio & Carvalho, 2013; Keysers, Kaas, & Gazzola, 2010). The role of the human mirror neuron system (hMNS), in empathy has been central to this debate (e.g. Bastiaansen et al., 2009; Decety, 2011; Gallese et al. 2004; Iacoboni & Dapretto, 2006). By serving to encode both the perception and expression of action used to communicate emotion, the hMNS (in humans this usually refers to premotor cortex and intra-parietal sulcus) potentially provides the foundation for empathic function. Zaki, Hennigan, Weber, and Ochsner (2010) suggest that the hMNS is engaged in empathic tasks when non-verbal social cues are involved. A review of some 200 functional magnetic resonance imaging (fMRI) studies (Van Overwalle & Baetens, 2009) found that the hMNS is engaged during perception and execution of articulated motions of body parts. They argued that this confirms the matching role of the mirror system in understanding biological action, and therefore, by implication, the actions of others. Some studies have associated neural activity or grey matter volume in the hMNS with empathic traits (Braadbaart, de Grauw, Perrett, Waiter, & Williams, 2014; Cheng et al., 2009; Pfeifer, Iacoboni, Mazziotta, & Dapretto, 2008; Lamm, Nusbaum, Meltzoff, & Decety, 2007; Schulte-Ruther, Markowitsch, Fink, & Piefke, 2007).

However, despite the appeal of this theory, most evidence has not provided support for a key role for systems serving action-representation in empathic function. Studies of empathic function during fMRI, and consequent meta-analyses of these studies (Fan, Duncan, de Greck, & Northoff, 2011; Lamm, Decety, & Singer, 2011) consistently relate empathy to activity of the anterior cingulate and insula, and evidence that action representation is involved has been somewhat lacking (Decety, 2011). A possible reason for this is the way that empathy is defined and conceptualized for the purpose of these meta-analyses. Empathy as defined by De Vignemont and Singer (see above) might also be termed “emotional contagion” (Hatfield, Cacioppo, & Rapson, 1993). Consequently, the emotions communicated are conceptualized as distinct from the actions which communicate them, rather than “embodied,” and experimental designs control for amount of action content. Most fMRI studies of empathy compare the influence of an action communicating an emotion or pain, with an action communicating something “neutral.” However, because the activity levels of brain areas serving action representation (whether at a somatosensory or programming level) are not influenced by the degree or type of emotion, it does not necessarily follow that they aren’t critical to the mechanisms for its transmission (Grèzes, Wicker, Berthoz, & de Gelder, 2009).

Empathy has also been defined as a capacity to understand other people’s feelings and to respond to them appropriately (Baron-Cohen & Wheelwright, 2004). Hence, attribution of motive or understanding (cognitive perspective-taking) is essential, for which purpose studies variously refer to false-belief tasks, metarepresentation, “theory-of-mind,” or inferential understanding. These studies implicate the temporo-parietal junction and ventromedial prefrontal cortex (Amodio & Frith, 2006; Saxe & Kanwisher, 2003; Zaki & Ochsner, 2012). Again, this perspective defines empathy as a cognitive process functioning independently of action representation.

Interestingly, those studies which have implicated the hMNS in empathy have associated it with individual differences in empathic traits rather than associating MNS activity with an empathic experimental condition. For example, both Braadbaart et al. (2014) and Pfeifer et al. (2008) found that activity in the MNS during facial imitation correlated with levels of empathic traits. Similarly, differences have been found in activity of the MNS between participants with and without autism spectrum disorder during an empathic task (Minio-Paluello, Baron-Cohen, Avenanti, Walsh, & Aglioti, 2009). With further respect to autism, the clinical literature finds that impaired empathic and nonverbal communication are closely related, insofar as they are both strongly associated with the diagnosis. Given increasing acceptance of the idea that autism might reflect a dimensional construct (e.g., Constantino & Todd, 2003), with traits being continuously distributed throughout typical populations, it follows that a similar relationship might exist in typical populations. Whilst mechanisms serving the perception and expression of action in social communication may not be specific to empathic function, individual differences in sensorimotor function may still be an important factor contributing to variability in expression of empathic traits in typical populations. Furthermore, variability in empathic traits may contribute to the development and maintenance of certain relevant sensorimotor functions.

Jackson and Decety (2004) refer to “motor cognition;” the fundamental unit of this paradigm being action, defined as “movements produced to satisfy an intention towards a specific goal, or in reaction to a meaningful event in the physical and social environments.” Motor cognition includes the processes involved in the perception, recognition, and interpretation of action as well as the processes concerned with action preparation and production. This would include those processes based in insula and somatosensory cortex serving the interoceptive representation of action-associated feelings. The question may therefore be posed as to what extent variability between individuals in levels of empathic traits is associated with variability in motor cognition within socioemotional contexts. And to what extent might dysfunction of mechanisms serving the perception and enactment of social actions occur in mental health problems such as schizophrenia and autism? On the perceptual side, the evidence points to only weak associations between reduced levels empathic traits and reduced capacity to identify emotions both in typical and clinical groups ( Dalton et al., 2005; Hadjikhani et al., 2004; Klin, Jones, Schultz, Volkmar, & Cohen, 2002; Law Smith, Montagne, Perrett, Gill, & Gallagher, 2010; Williams, Nicolson, Clephan, de Grauw, & Perrett, 2013), and indeed perception of emotional distress may even be enhanced in autism (Rogers, Dziobek, Hassenstab, Wolf, & Convit, 2007). Stronger evidence points to an important role of attention in determining the degree to which people with reduced empathy, such as those with autism or amygdala lesions, will attend to other’s actions (Dalton et al., 2005; Hadjikhani et al., 2004; Klin et al., 2002; Klin, Jones, Schultz, & Volkmar, 2003). With respect to the production of action, clinical studies of autism show that the expression of emotion and thought in action through gesture, eye movements, directed facial expression and modulated speech are strongly diagnostic (Lord et al., 2000; Rutter et al., 2000).

Nevertheless, the relationship between the *production* of non-verbal expression and empathy appears to be little studied, not least because of the difficulties of examining this in an fMRI environment. But there are also few tools available for examining individual differences in non-verbal expression in a purely behavioral context. For example, we would postulate that individuals who use a lot of gesture would also show a lot of facial expression, and would be more empathic, but we are not aware of any empirical evidence for this in the general population, or tools available for its study.

We considered that a self-report measure of motor cognition might constitute a simple initial step that could capture individual differences. More specifically, we considered that aspects of motor cognition concerned with the representation of actions communicating feeling states, would constitute an important aspect of empathy. We therefore sought to develop such a measure, and to test the hypothesis that (a) different aspects of motor cognition serving the communication of feelings (e.g., gesture, imagery, imitation) loaded onto a single construct, and (b) they correlated with empathic traits. If a single construct existed, we could examine possible neural substrates with participants who had recently taken part in study involving fMRI of facial imitation (Braadbaart et al., 2014).

## Study 1

### Methods

The study was approved by the University of Aberdeen Ethics Review Board for the College of Life Sciences and Medicine. A self-report questionnaire was devised that attempted to gauge people’s views on how much they imagined actions, read other people’s behavior, imitated other people, were susceptible to emotional contagion, and expressed thoughts and feelings through gesture, facial expression, and body posture. We wished to make the overall purpose of the questionnaire relatively opaque so as to minimize acquiescence bias. In doing this, we sought to make the questions quite neutral and also included questions that were marked negatively, though this was difficult without making questions sound awkward or convoluted. In the end, nine questions out of 30 were negatively scored. We called it the “Actions and Feelings Questionnaire” (AFQ), again to give it a neutral tone. Participants were asked to respond to the choices of “strongly agree,” “slightly agree,” “slightly disagree,” and “strongly disagree.” For positively scored questions, 3, 2, 1, or 0 points were given, respectively, in response to the statement “please tick the box that most applies to your view of yourself.” For negative questions, scoring was in the opposite direction.

A version of the questionnaire was first sent to a convenience sample of ten friends and colleagues to get some initial feedback about readability and ambiguity. The questionnaire was then modified according to suggestions and comments. It was then posted using an on-line survey instrument (Survey Monkey). Links to the survey were distributed by word of mouth, and through on-line social networks (Facebook) including the research group’s page. There were no exclusion criteria. The 30 questions that formed the AFQ were followed by 15 questions derived by Muncer and Ling (2006) from the Empathic Quotient Questionnaire (Baron-Cohen & Wheelwright, 2004) to make a short version. These were scored using the same options, but scoring followed a 2, 1, 0, 0 pattern as employed by the authors of that questionnaire.

In addition, questions were included at the beginning to ask about sex, year of birth, occupation (seven choices were given: “student,” “in employment or seeking employment,” “housework,” “retired,” “seeking work,” “retired,” or “other”), whether English was the first language, and country of residence. A statement was made to the effect that entrants should be over the age of 18 years. About 7 weeks after the original questionnaire was posted, an e-mail was sent to those participants who had provided their addresses to be sent further versions of the questionnaire. This email contained the link to the second version of the questionnaire. The second version was exactly the same as the first but the initial demographic questions and EQ questions at the end were omitted.

### Statistical analysis

The internal consistency of the AFQ was examined to gauge the extent to which items in the total scale give consistent responses. Two methods of measurement were applied: Cronbach’s alpha and item-total correlations. Conventionally, alphas between 0.7 and 0.9 and item-total correlations ≥0.3 are considered indicators of adequate internal consistency (Nunnally & Bernstein, 1994). As such, in this preliminary form of the scale, items with corrected item-total correlations ≥0.3 were retained in the questionnaire. Subsequent analyses were based on the scale constituting the retained items.

In order to identify the most appropriate number of factors to explain the pattern of co-variation in the variables of the AFQ, an exploratory factor analysis (EFA) was computed. A polychoric correlation was selected, as is appropriate where the distribution of ordinal items is skewed. An oblique rotation was selected, allowing for correlation between factors. The parallel analysis (PA) procedure of Horn was followed to determine the number of factors to retain (Horn, 1965), applying the optimal implementation of Timmerman and Lorenzo-Seva (2011). The intraclass correlation coefficient (ICC) and accompanying confidence intervals (CIs) were computed to express the between pair variance as a proportion of the total variance in the AFQ total score. The ICC varies between the values of 0 and 1 where 0 indicates no agreement and 1 indicates perfect agreement.

For each participant, a total AFQ score was calculated, as well as total scores for emerging factors. In view of the characteristics of the sample (see below), we investigated the effects of age, sex, and occupation on total and sub-total scores by dividing the group into two, and comparing the sub-groups with independent t-tests (male vs. female, student vs. other, and young vs. old – split according to median value). Age was also assessed with the rank correlation. Relationships with EQ were assessed using Pearson correlation coefficients.

Assessment of internal consistency, retest reliability, and convergent validity were assessed using SPSS Version 21. Parallel analysis was conducted using the FACTOR program (Davis, 1980; Gross & Barrett, 2011; Lorenzo-Seva & Ferrando, 2013).

## Results

### Participants

Of 318 registered entries to the questionnaire 278 valid responses were obtained. The remainder were inadequately completed. There were many more female compared to male respondents (189 females, 89 males; approximately a 2:1 ratio) but representation of males was almost exactly the same in older and younger age groups (32 % vs. 31.5 %). The population was also heavily biased towards a younger population and 119 (42.8 %) respondents reported themselves to be students. Those in employment or self-employed made up most of the remainder (144; 51.8 %) who described themselves as seeking work (n=3), engaged in housework (n=8), retired (n=10), and other (n=3). Only 12 (4.3 %) respondents did not continue school after minimum school age and 30 (10.8 %) participants said that English was not their first language. The younger population (n=128) had a mean year of birth of 1992 (age 21.2 years; SD 1.64; median 1993) whilst the older population (n=149) had a mean year of birth of 1969 (age 44 years; SD 11.7; median 1970). There was one missing value for age. The country of residence was the UK for 90 % of participants. The remainder came from elsewhere in Europe (n=17, 6 %) or North America (n=7), whilst one each came from Australia and Japan.

### Questionnaire properties

#### Internal consistency

Eighteen items had corrected item-total correlations ≥0.3 and were therefore retained to form the Final AFQ (see Table 1 for items). Reasons for low correlations for the 12 discarded items are unclear but could be because they were more sensitive to variance in attentional abilities, or because they asked about behaviors and experiences upon which people found it hard to reliably report. Internal consistency for the final questionnaire was robust with Cronbach’s alpha=0.81. All subsequent analyses were confined to these 18 items.

**Table 1:** Factor loadings* of the 18 item AFQ

**Item**	**Production**	**Perception**
1. I tend to pick up on people’s body language 2. To understand someone I rely on his or her words rather than their expression or gesture^§^ 3. To make sense of what someone else is doing, I might copy his or her actions 4. Music that I like makes me want to dance 5. In my mind's eye, I often see myself doing things 6. If talking on the phone, I am sensitive to someone's feelings by the tone of their voice 7. If others are dancing I want to join in 8. My body movements do not tend to reflect the way I feel^§^ 9. I often imagine myself performing common actions 10. I would consider myself to be a "touchy-feely" person 11. When I recall what someone said to me, I have to think hard to remember their facial expression at the time^§^ 12. I rely on seeing how a person looks me in the eye to gauge what they really feel 13. I wouldn't tend to know what someone was feeling like if they did not say^§^ 14. I move my hands a lot when I speak 15. I get animated when I am enthusiastic in conversation 16. I can easily bring to mind the look on someone's face when I remember telling them something 17. Acting things out helps me to understand them 18. Watching someone's body language is not a good way to judge their feelings^§^	−0.139 0.139 **0.518** **0.681** **0.377** −0.035 **0.646** 0.220 0.299 0.263 0.044 **0.303** −0.094 **0.340** **0.399** 0.021 **0.557** 0.148	**0.578** **0.362** −0.057 −0.100 0.269 **0.486** −0.010 0.279 0.190 **0.348** **0.631** 0.284 **0.502** 0.128 0.233 **0.640** 0.029 0.265

*Factor loadings >0.3 are shown in bold; ^§^Reverse scored items

#### Exploratory factor analysis

Item data were complete for 256 individuals. This number is sufficient for a reliable factor analysis and it is unlikely that a larger sample size would alter the analysis (MacCallum, Widaman, Zhang, & Hong, 1999). Parallel analysis identified a two-factor structure as giving the best explanation of the pattern co-variation. The first factor was composed of nine items and explained 23.8 % of the variance (Eigenvalue=4.29). The second factor was also composed of nine items and explained a further 10.7 % of the variance (Eigenvalue = 1.92). Table 1 shows the factor loadings on each item for the two factors. Cronbach’s alpha was 0.75 for the first subscale and 0.73 for the second.

Examination of the items contributing to the two factors showed that the items in the first factor were those that referred to production of action, and so we referred to this as the “production” sub-scale (e.g., “music that I like makes me want to dance to it” or “acting things out helps me to understand them”). Those in the second factor pertained to how people reported their experience of perceiving others’ actions (e.g., “I tend to pick up on people’s body language” or “when I remember what someone said to me, I often recall the look on their face”). We called this the “perception” sub-scale.

#### Test re-test reliability

**Fig. 1:**
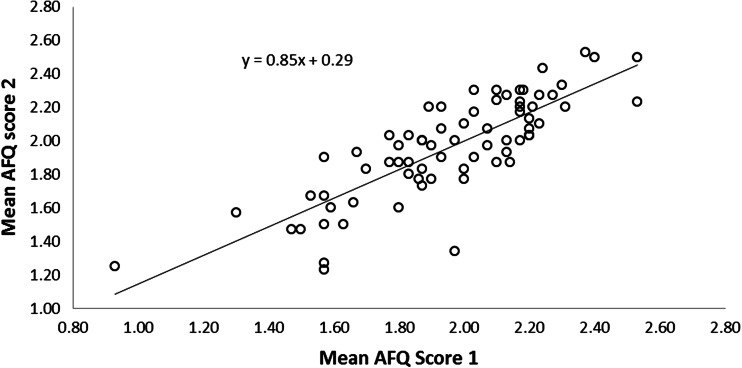
Scatterplot showing evidence for test retest reliability

Of 154 participants who consented to be sent the second version, 78 replies were received. Of these e-mail addresses did not correspond for four cases and two further cases did not provide valid AFQ scores. Therefore, Test-Retest data were available for 72 individuals. The ICC of the AFQ total score at the two time points was 0.88 (95 % CI=0.79 to 0.93) demonstrating acceptable retest reliability (see Fig. 1).

#### Individual differences and actions and feelings questionnaire scores

**Fig. 2:**
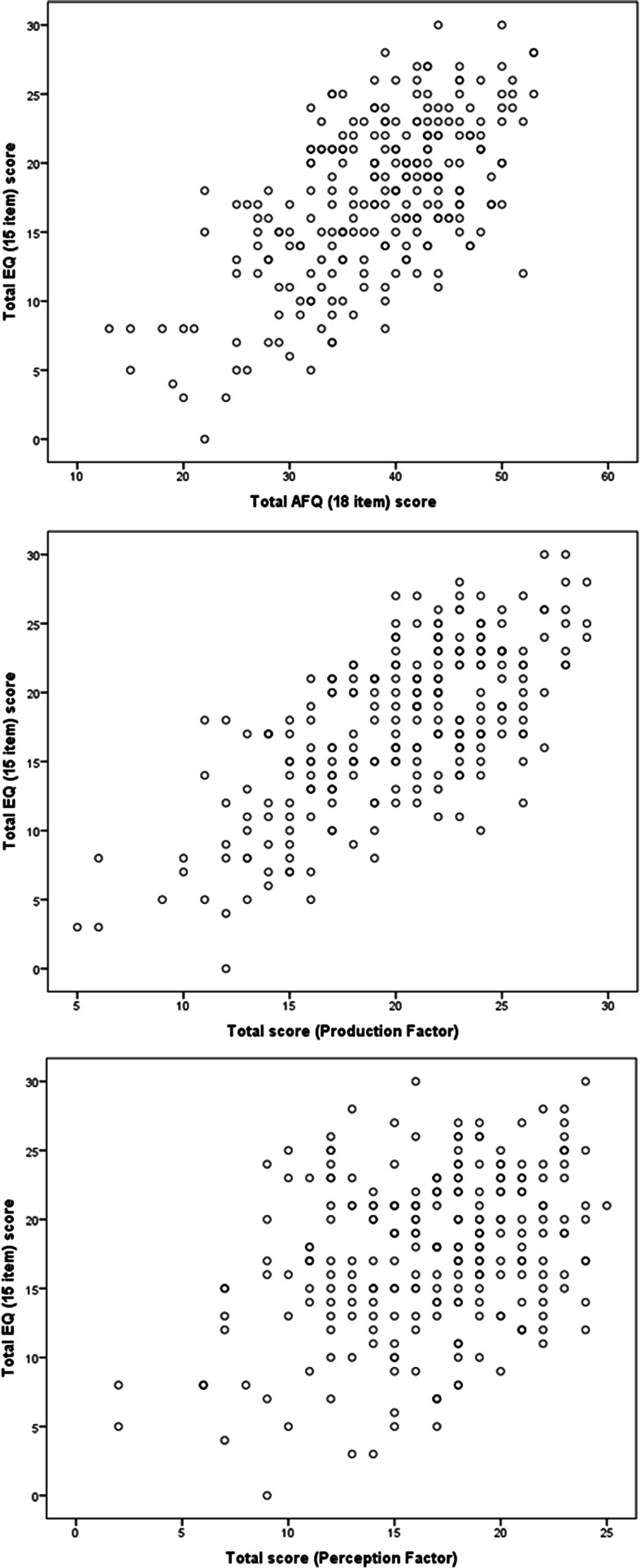
Scatterplots showing correlations between Total Actions and Feelings Questionnaire (AFQ) and factor scores with Empathic Quotient (EQ) total

The sum total scores and separate factors followed normal distribution curves (see Figs. 1 and 2). Overall the mean total score was 38.14 (n=257, SD=7.92) and sub-scale means were 20.5 (n=265, SD=4.63) and 16.7 (n=267; SD=4.45) for production and perception, respectively. Production and perception factor scores correlated with each other: r = 0.446, p<0.001. Measures for sub-groups are shown in Tables 2 and 3. These showed significant sex differences for total score and sub-scale scores, with a large effect size of >0.6 (Cohen’s d) similar to that found for the EQ (Baron-Cohen and Wheelwright, 2004). There was no significant difference between students and non-students. There was no correlation between total score and age (Spearman r = −0.037, p=0.552).

**Table 2:** Sex differences between measures

	Sex	N	Mean	SD	t	df	p	Mean Difference	Cohen’s d
Sum 18 item AFQ	M	83	34.61	7.63	−5.17	255	<0.0001	−5.20	−0.69
	F	174	39.82	7.51					
Sum Production	M	87	18.24	4.94	−4.61	148*	<0.0001	2.84	0.62
	F	178	21.08	4.17					
Sum Perception	M	85	15.39	4.13	−3.25	265	0.001	−1.87	−0.43
	F	182	17.26	4.48					
Sum EQ	M	84	15.04	5.70	−5.17	251	<0.0001	−3.78	−0.68
	F	169	18.82	5.37					

*Levene's test for unequal variance significant (p<0.04); equal variance not assumed.

**Table 3:** Mean group measures for age and occupation

	English 1st Language	N	Mean	SD	Age	N	Mean	SD	Student	N	Mean	SD
Sum 18 Item	Yes	229	37.96	7.72	Younger	133	38.56	7.66	Yes	114	38.19	7.99
	No	28	39.57	9.37	Older	123	37.58	8.14	No	143	38.09	7.88
Sum Production	Yes	236	20.05	4.56	Younger	138	20.42	4.54	Yes	117	20.15	4.65
	No	29	20.93	5.18	Older	126	19.80	4.71	No	148	20.14	4.63
Sum Perception	Yes	238	16.58	4.37	Younger	141	16.54	4.29	Yes	116	17.00	4.55
	No	29	17.31	5.15	Older	125	16.75	4.63	No	151	16.40	4.38
Sum EQ	Yes	224	17.34	5.72	Younger	133	18.28*	5.40	Yes	112	17.35	5.87
	No	29	19.28	5.85	Older	119	16.71*	6.05	No	141	17.73	5.68

All t-tests NS but for: *T=2.17(df=250), p=0.031

Robust correlations were found between the total EQ score, total AFQ score and between EQ and the two sub-scale scores (Total AFQ vs EQ: r= 0.615, n=257, p<0.0001; Production vs EQ: r=0.689, n=240, p<0.0001; Perception vs EQ: r=0.347, n=247, p<0.0001; see Fig. 2). As the correlation between Production and EQ was stronger than that between EQ and both the total score and Production, we ran a linear regression to examine the relative contribution of the two sub-scales to the relationship. This showed that after controlling for the Production score, the correlation between Perception and EQ was non-significant (Production: Standardized Beta=0.673, t=12.93, p<0.0001,Beta 95 % CI=0.71–0.96; Perception: Standardized Beta=0.051, t=0.98,p=0.33, Beta 95 % CI=−0.07–0.20)

#### Discussion (study 1)

We sought to design a self-report measure that would capture individual differences in experiences of cognitive reliance on action. Our results suggest that we achieved this aim. Our final questionnaire consisting of 18 items, demonstrated highly satisfactory internal consistency and rate-rerate reliability. Total scores were uninfluenced by age or occupation, but showed a marked effect of sex, with an effect-size of a very similar magnitude, and in the same direction, to that found with the EQ questionnaire (Baron-Cohen & Wheelwright, 2004).

Scores were also uninfluenced by whether English was a first language providing an indicator that they are not highly dependent upon language skills. We also found a robust correlation with the 15-item EQ, suggesting that both questionnaires tap into common underlying traits.

Of further interest were the two factors that emerged from the exploratory factor analysis. The first factor,which we have called “Production,” was loaded with items that asked about the expression of thought and feelings through action, or imagining acting-out. The second factor was loaded with items that asked about perceptions and memories of other’s actions (especially others’ expressions of emotions) and so we have called this “Perception.” Whilst both factors and the total score all correlated with the EQ score, the correlation of “Perception” with EQ was weaker and became non-significant after controlling for “Production.” Similarly, the effect of sex for “Perception” was weaker than that for the “Production” factor. If the association between AFQ and EQ scores was simply mediated by a tendency to rate one’s self as more sociable, emotionally sensitive or reactive, we would have expected the opposite, since five out of six items that used the words “feel,” “feelings,” or “feely” loaded more onto the Perception factor.

As mentioned in the introduction, the EQ is thought to be driven by quite a “cognitive” definition of empathy – relying on a capacity to understand others feelings and make appropriate judgments as to how to respond to them. This raises the question as to whether “perception” and “production” factors could relate differentially to “affective” and “cognitive” aspects of empathy. Further work might explore the relationship between the AFQ and the Toronto Empathy Questionnaire (TEQ), which is designed to tap into more emotional aspects of empathy (Spreng, McKinnon, Mar, & Levine, 2009), in which case we may predict these associations to be reversed. However, we note that in a large study of the EQ, evidence was not found for separate cognitive and affective components of empathy (Allison, Baron-Cohen, Wheelwright, Stone, & Muncer, 2011). Furthermore, the EQ correlates highly with the TEQ (r=0.8, n=65; Spreng et al., 2009). Therefore, it remains unclear as to whether cognitive and emotional empathic traits can be discerned from one another as separate sources of individual differences. In addition, in our questionnaire those items which one would have been predicted to tap more onto an “affective” or “emotional contagion” factor (such as “music I like makes me want to dance”), actually loaded more heavily on to the “production” factor. We note that the two-factor model derived from this study still requires confirmation and the model therefore still requires assessment in further samples using confirmatory factor analysis.

One pertinent observation is that the AFQ concerns itself with self-report of personal experience, and therefore with the degree to which individuals are self-aware of both perceived and enacted action. It is therefore possible that it reflects levels of action-awareness more than actual dependence on nonverbal cognition. We anticipated that study 2 might throw some light on this hypothesis and to help to explain the relationship between our measure and empathy.

## Study 2

### Background

We recently conducted a study investigating the neural correlates of facial imitation accuracy (Braadbaart et al., 2014), in which volunteers were asked to imitate a range of facial expressions. The expressions were created by morphing basic emotional expressions in varying amounts, to create arrays in which many expressions were closely similar to one another, thus creating an imitation task which required participants to copy emotion-conveying expressions that were as accurate as possible. The task differs from other facial imitation tasks in placing high demands on the capacity for control over the facial expression of emotion. We have repeatedly found that performance on the task correlates with empathic quotient (Braadbaart et al., 2014; Williams et al., 2013). In the fMRI study, we compared activity during the imitation task with a control condition, where participants executed a predetermined facial action according to instruction. EQ correlated with the level of activity during imitation relative to that during response to instruction in intraparietal sulcus, dorsal premotor cortex, somatosensory cortex, and hippocampus, providing evidence that greater activity of sensorimotor systems during imitation is associated with higher levels of empathic traits. We hypothesized that the relationship between AFQ score and EQ might also be mediated by common reliance on these regions.

### Methods

A more detailed description of the Facial imitation task and fMRI study can be found in Williams et al. (2013) and Braadbaart et al. (2014). Twenty right-handed participants (ten female) between 19 and 45 years old were asked to lie in a 3 T MRI-scanner (Achieva X-series, Philips Medical, Best, The Netherlands) with a 32-channel phased-array head coil (technical parameters in Braadbaart et al., 2014). After initial structural scans, the fMRI study consisted of just two conditions. Participants were shown manipulated male and female face stimuli showing expressions of the six basic emotions, either pure or blended in variable amounts. A letter above the screen provided instruction: an “I” indicated a requirement to Imitate, an “O" to form an “O” with the mouth, and a “T” to stick out the tongue. This control condition was referred to as an execution Mismatch as the same expressions were shown for both “Imitate” and “Mismatch.” Performance was monitored using a camera mounted to the head coil. After pre-processing the data, we investigated differences in activation between Imitate and Mismatch.

For the purposes of this study, permission was sought from the local ethics committee to trace participants and invite them to complete the AFQ. Of the original 20 participants, valid responses were obtained from 12 participants (mean age: 25.9 years [SD=5.52], six females). From the replies, total AFQ score and scores for the two factors were calculated and we tested for correlations first with the other behavioral measures of performance, and then with the difference in BOLD response between Imitate and Mismatch using SPM. Threshold for statistical significance was determined at a cluster level of 38 voxels at p<.001, derived from Monte Carlo simulations (Forman et al., 1995; Slotnick, Moo, Segal, & Hart, 2003).

### Results

**Fig. 3:**
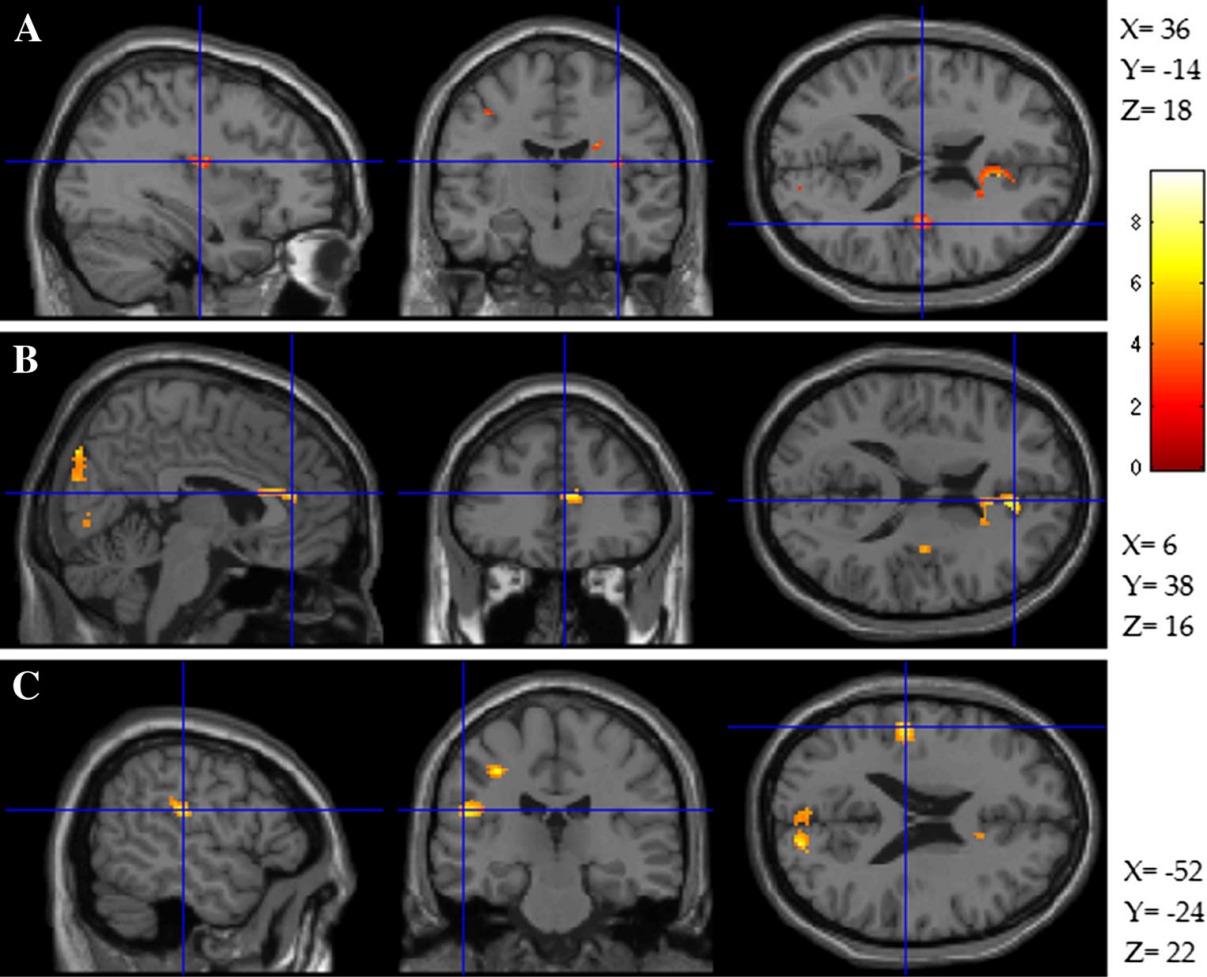
Slices demonstrating locations of significant correlation between Total Actions and Feelings Questionnaire (AFQ) score and contrast of imitation vs. instruction in the (**A**) insula, (**B**) anterior cingulate, and (**C**) somatosensory cortex as described in Table 2

Results are shown in Table 4 and Fig. 3. We found no significant correlations between EQ and either total score or the two subscale scores (unsurprising given the small sample size). Looking at the fMRI data, we found significant positive correlations between total AFQ and activity during Imitation compared to Mismatch in the sensorimotor cortex, insula, and anterior cingulate, and the visual cortex. The subscales showed broadly similar relationships but the Production factor also correlated with caudate activity. All three scales also showed negative correlations with activity in the Fusiform gyrus.

**Table 4:** Brain areas where contrast of activity between Imitation and Mismatch conditions correlated significantly with AFQ Total score and factor scores (statistical threshold determined at a cluster level of 38 voxels at p<.001, derived from Monte Carlo simulations)

	Location	Cluster Size	T	Z-score	X	Y	Z
Total Score (positive)	Lingual	129	9.6	4.73	24	−72	−10
	Lingual	319	9.04	4.61	−22	−76	0
	Occipital	348	7.88	4.35	16	−84	22
	Anterior cingulate	124	7.1	4.15	10	38	16
	Somatosensory	125	6.97	4.11	−52	−24	22
	Postcentral gyrus	85	6.92	4.1	−34	−24	46
	Somatosensory	41	6.1	3.85	28	−10	30
	Insula	38	5.39	3.61	36	−14	18
Total score (negative)	Fusiform	77	7.5	4.26	42	−36	−18
	Cerebellum	66	5.68	3.71	8	−34	−20
	Precentral	64	5.47	3.64	28	−20	56
Factor 1 (positive)	Lingual	87	7.24	4.19	−8	−64	−6
	Caudate	131	6.99	4.12	−26	0	24
	Postcentral	108	6.85	4.08	−36	−16	44
	Angular	89	6.64	4.02	−34	−70	36
	Insula	40	6.03	3.83	36	−12	18
	Cuneus	169	5.7	3.72	10	−80	38
Factor 1 (negative)	Fusiform	91	9.26	4.66	42	−36	−20
Factor 2 (positive)	Lingual	55	7.42	4.24	12	−76	−6
	Lingual	150	6.54	3.99	−16	−80	−4
	Operculum/supramarginal	41	6.44	3.96	−50	−24	20
	Postcentral	48	6.3	3.92	−60	2	22
Factor 2 (negative)	Inferior frontal	86	10.07	4.81	48	22	10
	fusiform	52	6.27	3.91	44	−34	−16
	precentral	49	6.16	3.87	28	−18	54
	mid temporal	43	5.74	3.73	−64	−40	10

### Discussion

The results of this analysis revealed a relationship between AFQ total score and activity in the network of somatosensory cortex, insula, and anterior cingulate during imitation compared to an instruction condition. As discussed above, whilst there is widespread acceptance for the role of anterior cingulate and insula in empathy, the role of somatosensory cortex is less clear. fMRI studies suggest that the somatosensory cortex serves a role for vicarious perception of touch but not emotion (Keysers et al., 2010; Molenberghs, Cunnington, & Mattingley, 2012; Zaki et al., 2010). In a meta-analysis, Lamm et al. (2011), found that the insula and anterior cingulate were consistently associated with empathy, but that the somatosensory cortex was involved non-specifically, and only when strong visual cues were involved.

However, Straube and Miltner (2011) carried out a study in which they parametrically increased the degree to which participants were required to attend to the emotional content of the stimuli and their own reaction. Attention to emotional content and own experience was associated specifically with increasing activity in somatosensory cortex and posterior insula. Straube and Miltner suggest that somatosensory cortex may be important for attending to own feelings and inferring emotional intensity from that experience. Earlier work has also implicated the somatosensory cortex in mediating attention-to-action (Rushworth, Krams, & Passingham, 2001). In two other studies, somatosensory cortex activation was associated with emotion appraisal during an empathic task (Lamm, Nusbaum, Meltzoff, & Decety, 2007) and with judgment about an action in a perspective-taking task (Lamm, Fischer, & Decety, 2007). Adolphs, Damasio, Tranel, Cooper, and Damasio (2000) found that impaired recognition of facial emotion followed lesions to somatosensory cortex and Pitcher, Garrido, Walsh, and Duchaine (2008) found that transcranial magnetic stimulation (TMS) to somatosensory cortex disrupted emotion recognition. These authors suggest that somatosensory cortex is involved in the recognition of emotion by simulation of feeling states that correspond to those perceived. In this study, in the contrast between imitation and the control condition, it was precisely such a simulation function that was being contrasted.

Furthermore, Picher et al. (2008) also found that TMS to the lingual gyrus disrupted emotional recognition. The correlations that we also found in visual cortex could therefore possibly reflect the role of this region in emotional simulation, but could also reflect a difference in the amount that participants were looking at the facial stimuli in the two conditions. In the imitation condition, participants were required to look carefully at the face in order to imitate it accurately. In the instruction condition, the facial stimuli were the same but played no meaningful role in the task. Therefore, it may be that the correlations stem from differential amounts of attention being paid to the faces in the two conditions and, therefore, that the AFQ is predicting the differential amount of attention being paid to the face for the purpose of imitation compared to when just the instruction was followed. The ventromedial prefrontal cortex is also thought to play a role in mediating attention to faces (Wang, Ramsey, & Hamilton, 2011; Wolf, Philippi, Motzkin, Baskaya, & Koenigs, 2014). Together therefore, the pattern of activity in this analysis suggests that the AFQ score might correlate with activity that reflects the degree to which participants attended to the facial stimuli and also their own responses to them during an imitation task. The negative correlation with activity in the face fusiform area might be explained if looking to imitate emotional expressions somehow disengaged other face processing in fusiform face area.

## General discussion

Our first study hypothesized that a common construct would underpin a range of self-reported socioemotional behaviors that were all reliant on the perception, imagination, or production of action, and furthermore that a measure on this construct would correlate with self-reported empathy. Our first study revealed evidence for a coherent construct that had good test-retest reliability, indicating that the measure reflected normally distributed, between-individual variation that remained stable over a period of about 7 weeks. Also, the construct correlated well with EQ. On reflection, we considered that because the questionnaire asked participants to report on their experience, it may provide a measure of self-awareness of action.

It might be suggested that the correlation between EQ and AFQ arose because the content of the questionnaires overlaps. However, the emphasis of the EQ is on social understanding, judgment, motivation, and reactivity to expressions of emotions, whilst in contrast, the AFQ asks respondents to report self-awareness of their own and other people’s actions. Some overlap may occur where EQ questions are concerned with awareness of other’s behavior, or some AFQ items ask about emotional reactivity when referring to awareness of others. This seems insufficient on its own to account for the correlation which suggests a stronger association between the constructs.

In itself, a strong correlation between self-report levels of non-verbal behavior and self-report of empathic traits is significant. Much research has drawn a distinction between mechanisms serving action representation and inferential reasoning (Zaki and Ochsner, 2012), or been concerned with identifying neural substrate specific to empathy which does not include brain areas serving action-programming. However, this does not preclude the possibility that individual differences in mechanisms serving action representation are not important for generating variability of expression of empathic traits in the population as a whole. Our finding suggests that this is indeed the case, though because we note that this is a self-report measure, it may to some degree reflect a capacity for introspection and self-awareness. Therefore, our behavioral findings suggest that self-awareness of feelings expressed in actions is an important component of empathy. Self-awareness of actions relies on a capacity form a “secondary” representation of action that is distinct from the primary coding of that action in either perceptual or motoric modalities (Suddendorf & Whiten, 2001) and this is likely to be necessary for both perspective-taking (Lamm et al., 2007) and differentiation between self and other (Decety 2011)

The fMRI analysis informed the findings of the questionnaire study. The rationale behind this hypothesis was that the facial imitation task we employed was designed to elicit carefully controlled enactment of socioemotional behavior, and would therefore place high demands on those brain mechanisms utilized for the perception and enactment of emotional expression. We expected that this task would be sensitive to individual differences in activity of cognitive mechanisms mediating the representation of social actions. The total score correlated with activity in the circuit consisting of anterior cingulate, somatosensory cortex, and insula, but also visual cortex, was consistent with activity reflecting attention-to-action and action-awareness.

These findings appear to be in accord with the behavioral findings. They suggest that variability in the distribution of empathic traits within typical populations is heavily influenced by the capacity to attend to one’s own internal representations of actions associated with feelings. Presumably, this capacity is critical to the self-awareness of one’s own feeling states, which in itself is critical for empathy. Not only do we see a strong correlation in this capacity with EQ but also marked sex differences.

However, this does beg the question of why we found a stronger association between EQ and questions that concerned themselves with the production rather than the perception of action. As mentioned one possibility is that both the “production” factor and the EQ are oriented more towards “cognitive” aspects of empathy, but the question remains as to why they should be related. One possible explanation is provided by the Enactive Mind (EM) framework (Klin et al., 2003) which seeks to “highlight the central role of motivational predispositions to respond to social stimuli and a developmental process in which social cognition results from social action”(p.348). Within this framework, the focus is on the mechanisms by which individuals engage in the processes of acquiring social competences in the first place. A mechanism that fosters attention to relevant action-cues for the purpose of enacting appropriate responses is central to the development of social cognition, and an inclination to attend to action would develop into higher levels of action-awareness, which is then detected as a trait measure by our Actions and Feelings Questionnaire. In our fMRI study, AFQ score appears to be correlated with the effects of extra attention being paid to the face when extra attention was required to complete the task as well as possible, as well as activity within the empathic network driving attentional differences. According the EM framework, in the context of this fMRI study, the AFQ total score is providing a measure of the degree to which attentional mechanisms are adapted to attend to action for the purpose of imitating it. In the broader context, this would suggest that the degree to which attention mechanisms are adapted to attend to action-cues can be measured using the AFQ and that this measure is associated with levels of empathic traits.

Our stronger correlation between EQ and the production subscale as opposed to the perception subscale is consistent with the EM framework, in that the former subscale reflects not just attention to others’ actions, but attention to others’ action for the purpose of enactment (as also occurred during the facial imitation task). Therefore, it appears to be the capacity of the system to attend to those action-cues that are relevant to own intentions that determines an association with both empathic traits and AFQ.

As noted we found marked sex differences in our questionnaire comparable to those seen with the EQ. This generates the hypothesis that there will be sex differences in a capacity to attend to relevant action-cues. Whilst we are not aware of research specifically identifying sex differences in attention-to-action, there is evidence that women show a higher degree of emotional awareness than men (Barrett, Lane, Sechrest, & Schwartz, 2000). The presence of sex differences therefore add further support to the suggestion that this questionnaire taps into individual differences in self-awareness.

One limitation of the questionnaire study is that the sample was not representative of a typical human population, being largely British, and involving disproportionate numbers of females, students, and young people. Our initial findings do not suggest that age or background has a significant effect on score, but these require confirmation with a more representative sample. Also, we have only explored the association between scores on this questionnaire and a single measure of empathic traits. In future research it will be of interest to explore associations with other measures of empathic function and emotional awareness. Clearly, the correlational study employing the fMRI data is based on small numbers and requires replication with a larger sample.

In summary, in an initial study with this novel self-report questionnaire, we found evidence for a coherent and reliable measure of action-awareness that corresponds to individual differences in empathic traits, reveals marked effects of sex and suggests that mechanisms controlling attention to action and self-awareness of action may be an important source of individual differences in empathy. We suggest that a capacity or willingness to attend to one’s own internal representations of actions which serve feeling states, constitutes an important source of variability in the expression of empathic traits in typical populations. It is a commonly held belief that “touchy-feely” types of people are more empathic. Our research suggests that this might be a particularly apt term. We note the comment made by Decety (2011) that the construct of empathy “may be too complex to be both meaningful and useful for sound research in affective and social neuroscience,” and that breaking it down into component processes will be beneficial in the exploration of psychiatric disorders or abnormal empathy. The AFQ may promise to be a useful tool in this endeavor by targeting a specific aspect of empathic functioning, i.e. bodily awareness and expression, that is critical to social behavior and disrupted in a range of mental health problems.
